# Using musical pitch interval comparisons to assess cochlear implant frequency-to-place maps

**DOI:** 10.3389/fauot.2025.1565883

**Published:** 2025-05-18

**Authors:** Rebecca M. Lewis, Melanie L. Gilbert, Jordan A. Beim, Andrew J. Oxenham, Charles J. Limb

**Affiliations:** 1 Department of Otolaryngology-Head and Neck Surgery, University of California, San Francisco, San Francisco, CA, United States,; 2 Department of Psychology, University of Minnesota, Minneapolis, MN, United States

**Keywords:** vocoded stimuli, flat panel computed tomography (FPCT), frequency-to-place mismatch, musical pitch intervals, cochlear implant programming, pitch perception, cochlear implant, normal hearing

## Abstract

**Introduction:**

Music perception remains challenging for many cochlear implant (CI) recipients, due perhaps in part to a frequency mismatch that can occur between the original tonotopic cochlear map and the allocation of frequencies along the electrode array that occurs during programming. Individual differences in ear anatomy, electrode array length, and surgical insertion can lead to great variability in the positions of electrodes within the cochlea, but these differences are not typically accounted for by current CI programming techniques.

**Objectives:**

Flat panel computed tomography (FPCT) can be used to visualize the location of the electrodes and calculate the corresponding spiral ganglion characteristic frequencies. Such FPCT-based CI frequency mapping may improve pitch perception accuracy, and thus music appreciation, as well as speech perception. The present study seeks to develop a behavioral assessment metric for how well place-based pitch is represented across the frequency spectrum by evaluating the accuracy with which listeners perceive and compare pitch intervals across different frequency regions.

**Methods:**

The study included two groups: normal-hearing (NH) listeners and CI recipients. Listeners were asked to match the pitch interval created by two tones, played sequentially, across different frequency ranges to estimate the extent to which pitch is evenly distributed across the CI array. This test was initially evaluated with pure tones in normal-hearing listeners, using both unprocessed and vocoder-processed sounds to simulate both matched and mismatched frequency-to-place maps. We hypothesized that the vocoded stimuli would be more difficult to match in terms of pitch intervals than unprocessed stimuli, and that a warped map (as may occur with current clinical maps) would produce poorer matches than a veridical and well-aligned map (as may be achieved using FPCT-based frequency allocation).

**Results:**

Preliminary results suggest that the task can reveal differences between veridical and warped maps in normal-hearing listeners under vocoded conditions. A small cohort of CI recipients were tested with the same pure tone stimuli (without vocoding). Performance of the CI recipients was similar to that of normal-hearing listeners, and both groups showed less accurate interval matching compared to NH listeners.

**Discussion:**

The results suggest promise for this method when comparing the perceptual effects on pitch interval perception of traditional clinical maps and FPCT-based frequency allocation.

## Introduction

Cochlear implants (CIs) provide individuals who have severe-to-profound hearing loss and poor word recognition the opportunity to regain sound awareness, improve speech understanding, and significantly improve their quality of life (Contrera et al., 2016; Johnson et al., 2024; Low et al., 2020; Sousa et al., 2018). Throughout CI development, the primary focus has historically been on optimizing speech perception, particularly in quiet environments. Through the last several decades of research, CIs have demonstrated their efficacy in facilitating speech comprehension under such conditions (reviewed by Boisvert et al., 2020; Ruben, 2018). However, their performance in more complex auditory domains, such as music or speech in noisy environments, often falls short of patient expectations (Abdulbaki et al., 2023; Fabie et al., 2023; Fowler et al., 2021; Limb and Roy, 2014). This shortfall highlights a critical challenge in implementing CI technology – while CI recipients can often achieve impressive speech understanding scores in quiet, the perceived sound quality can be far from ideal (Berg et al., 2023; Kurz et al., 2023), also affecting the ability to accurately perceive emotion of the voice (Barrett et al., 2020; reviewed by Jiam et al., 2017) and the speaker’s identity (Mamun et al., 2019, 2023). In the same way, clinicians note that two patients using maps that produce similar speech-in-quiet test results (or a patient using two different maps) may experience vastly different sound qualities (Dorman et al., 2024), which may affect their music perception without affecting their speech recognition scores.

Music perception remains challenging for many CI recipients, likely due to the inherently poor spectral resolution of CIs (e.g., Oxenham, 2023), but also perhaps in part to the frequency mismatch that occurs between the electrode placement along the cochlea and the frequencies allocated to each electrode in the CI map, or the listener’s natural tonotopic organization (Jiam et al., 2019b, 2021; Kurz et al., 2023). Interpersonal and interaural differences in inner ear anatomy (Hrncirik et al., 2022; Hussain et al., 2023), electrode array length (Canfarotta et al., 2021; Thompson et al., 2023), and differences in surgical insertion (Högerle et al., 2022; Hrncirik et al., 2022; Kant et al., 2022) can all contribute to substantial variability in the positions of electrodes within the cochlea, but these differences are not typically accounted for by current CI programming techniques. Using Flat Panel Computed Tomography (FPCT), the position of the CI electrodes can be determined, allowing for characteristic frequencies (CFs) for a healthy ear to be predicted from that CI electrode position (Jiam et al., 2019b, 2021). Previous studies have explored the use of FPCT-based mapping to improve speech perception, demonstrating that frequency allocation can lead to improvements in speech intelligibility (Jaekel et al., 2017; Park et al., 2012; Shader et al., 2020; Zinfollino et al., 2020). However, its impact on music and pitch perception remains largely unexamined, despite the critical role of accurate pitch processing in auditory experience beyond speech.

There are two ways in which an inaccurate frequency-to-electrode map might affect place-based pitch perception: first, it may create a mismatch between the actual and expected absolute pitch that is based on a normal, acoustic cochlear map (Goupell et al., 2019). Second, it may distort pitch relations, i.e., the musical interval created between two frequencies. Given that relative pitch tends to play a much more important role in music than absolute pitch (Hutchison et al., 2017; Moulton, 2014), this second aspect may be particularly critical to improving the perception of pitch and appreciation of music in CI recipients (Landsberger et al., 2022). Further, perception of musical intervals as equal logarithmic frequency sizes is inherent even across very different cultures (Jacoby et al., 2019), which underscores the importance of ensuring that the frequency mapping for CI users aligns with these universal principles of pitch perception, potentially enhancing not only music perception but also the broader auditory experience.

This study builds on previous research by specifically investigating the perceptual effects of anatomically based remapping on musical pitch intervals in normal-hearing listeners, an area that has been largely unexplored. While prior studies have examined the impact of remapping on speech understanding, the question of whether such remapping preserves or improves pitch interval accuracy remains unanswered. To address this, we introduce a novel behavioral task designed to assess pitch interval perception across different frequency regions in CI users, validating it in normal-hearing listeners while simultaneously piloting it to ensure usability and relevance among CI users.

To address the question of relative pitch perception, the present study was designed to create and validate a novel test, termed the Musical Pitch Interval Comparison Task. The goal of this test is to determine whether the perceived size of musical intervals (i.e., pitch differences between two tones) is preserved across the frequency range utilized by the electrode array in CI recipients. By directly assessing pitch interval accuracy, this study provides a behavioral framework for evaluating how frequency-to-place alignment affects musical pitch perception and informs future CI programming strategies that extend beyond speech.

As an initial validation of the test, we studied the performance of three groups of participants: (1) normal-hearing (NH) listeners, who completed the task with pure-tone (non-vocoded) stimuli, (2) NH listeners who completed the task with the pure tones passed through three different vocoder configurations, designed to represent maps with which CI recipients could be fit, and (3) CI recipients using a map with the manufacturer’s default filterbank.

## Methods

### Participants

This study involved NH listeners and CI recipients. The NH listeners provided demographic and music training history data for comparison, though one participant did not provide age and another did not provide music training experience. A small pilot group of CI recipients was tested to determine the approach’s feasibility without extensive training before data collection began.

#### Normal-hearing participants

Thirty-one adults were recruited through the University of Minnesota for online testing, reporting normal hearing. The NH group that assessed unprocessed stimuli had 15 participants (average age 22.6 years, SD ± 1.5; 5 males, 10 females) with 8.1 years (SD ± 3.9) of musical training. The NH group that assessed vocoded stimuli had 16 participants (average age 28.6 years, SD ± 13.8; 7 males, 9 females) with 11.1 years (SD ± 11.1) of musical training. Both NH groups completed testing remotely via an online MATLAB (Mathworks Inc., Natick, MA) platform using headphones.

#### Cochlear implant recipients

Nine CI recipients (Table 1, average age: 57.4 years, SD: ±13.2; 6 males, 3 females) were recruited through the University of California, San Francisco. This group consisted of one bilateral and eight unilateral CI recipients, all equipped with MED-EL CIs and using their clinical everyday listening programs, which utilizes the manufacturer default logarithmic frequency allocation table (i.e., LogFS). Their reported musical training averaged 11.3 years (SD: ±12.3). Similar to the NH group, the CI cohort completed the task via an online MATLAB platform. CI recipients were instructed to use their preferred home transducer—soundfield speakers, headphones, or streaming—ensuring the test ear was isolated.

### Pitch interval assessment procedure

We focused on pitch interval comparisons across the frequency range of contemporary CI processors, divided into low (root note 150 Hz; interval range 126–505 Hz), mid (572 Hz; 480–1,924 Hz), and high (2,181 Hz; 1,833–7,314 Hz) regions. Participants were presented with two melodic pitch intervals between frequency regions in succession, and were asked to identify the larger interval (McDermott et al., 2010; Zarate et al., 2012). Each pitch interval consisted of a 3-tone melody (low-high-low sequence) with the first and last notes the same (e.g., C5-G5-C5). The melody’s root note frequency was roved within a half-octave range, centered around its nominal frequency and the three 300-ms pure tones (gated with 30-ms onset and 50-ms offset raised-cosine ramps) were separated by 150-ms gaps. For NH listeners assessing unprocessed pure tones, the fixed interval was either 4 or 7 semitones (ST; these intervals correspond to a major third and perfect fifth, respectively), while for CI recipients and NH listeners with vocoded stimuli, it was always 7 ST. Intervals used equal temperament tuning, with 1 ST representing a change in frequency (cycles per second) by a factor of 2^(1/12)^.

#### Adaptive tracking procedure

The assessment employed an adaptive testing approach (e.g., Jesteadt, 1980) to determine each participant’s point of subjective equality (PSE) for the size of a pitch interval, compared across two distinct frequency regions. One of the two intervals was fixed, and the other interval was adaptively varied, based on the listener’s previous responses. The size of the adaptively varying interval is quoted relative to the size of the fixed interval, so that a value of 0 ST indicates that the adaptively varying interval was the same size as the fixed interval (either 4 or 7 ST).

Each run consisted of four randomly interleaved adaptive tracks: two used a 2-down 1-up procedure (tracking the 71% point of the psychometric function; Levitt, 1971) and two used a 1-down 2-up procedure (tracking the 29% point of the psychometric function). One pair of tracks varied the first (lower) interval and the other varied the second (higher) interval. The varying interval’s starting size was randomly chosen within ±3 ST of the fixed interval. The initial step size of 4 ST decreased to 2 ST after the first two reversals. Each track terminated after six reversals, with the threshold calculated as the mean of the last two reversals. Trials from terminated tracks were randomly interspersed with trials from the remaining tracks but were not recorded.

Once all tracks had terminated, the PSE was defined as the average of the four tracks (since the mean values at the 71% and 29% points approximate that at the 50% point). Each participant completed five runs per condition, with prompts to rest between runs. The adaptive tracking was limited to values between−7 and +10 ST. If a track exceeded these limits more than six times, it was terminated and assigned a value of−8 or +11 ST, respectively. Participants completed a short training module using 7 ST intervals, with feedback, before data collection, but no feedback was provided during testing.

#### Stimuli

The NH participants were assessed using pure-tone stimuli that were either unprocessed or passed through a noise-excited envelope vocoder to simulate aspects of CI sound processing. The CI cohort was presented with only unprocessed pure-tone stimuli during the assessment.

Only the Mid:High frequency range comparisons (i.e., 480–1,924 Hz to 1,833–7,314 Hz) were used with the CI recipients and the NH listeners under vocoding, since the low frequency range (126–505 Hz) was not accessible to the vocoded condition modeling the shorter electrode array, where the most apical simulated channel had a CF of 500 Hz.

**FIGURE 1:**
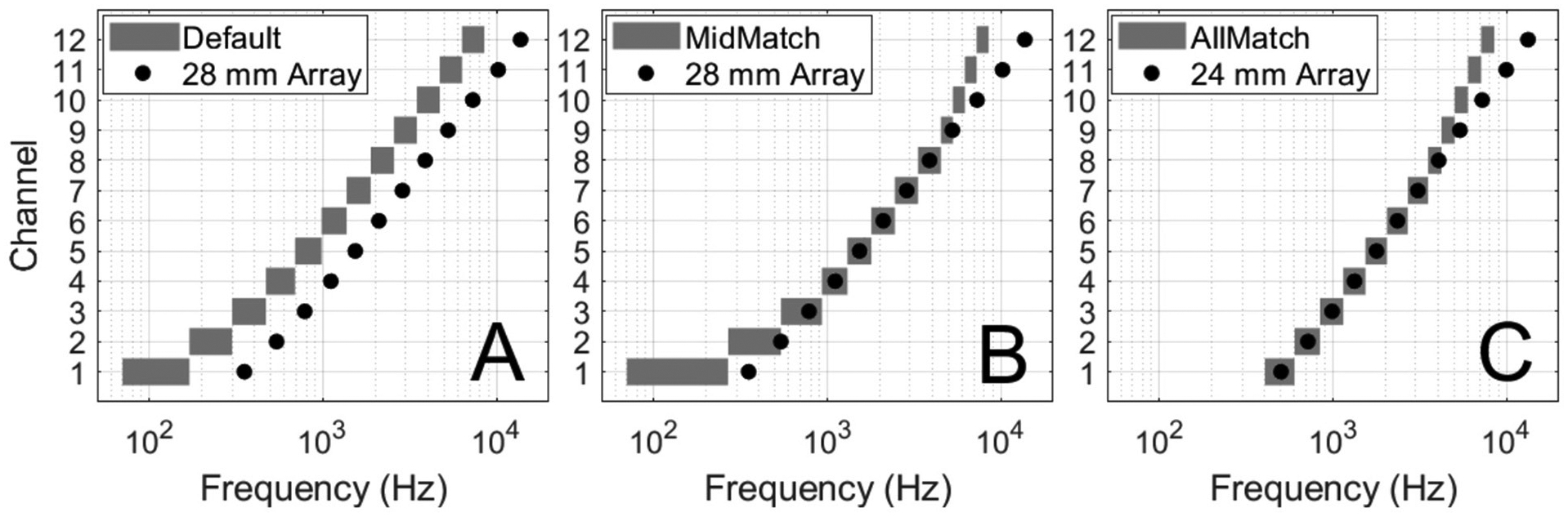
Frequency allocation tables for vocoded conditions. CI frequency allocation tables (horizontal bars) with electrode locations (circles). **(A)** The 28 mm electrode array with the CI manufacturer’s default logarithmic filterbank (LogFS, “Default”). **(B)** The 28 mm electrode array with middle frequencies (950–4,000 Hz) matched (“MidMatch”). **(C)** The 24 mm electrode array with all frequencies (<4,000 Hz) matched (“AllMatch”).

To create the vocoded stimuli, a frequency warp was applied to simulate either a full length (28 mm) or a shorter (24 mm) electrode array placement, based on spiral ganglion CFs derived from previous FPCT-imaged cochlear duct measurements (Helpard et al., 2020, 2021; Jiam et al., 2021; Li et al., 2021). The most apical electrode of the 28 mm array corresponded to a CF of 350 Hz (Figures 1A, B), and the most apical electrode of the 24 mm array corresponded to a CF of to 500 Hz (Figure 1C), consistent with larger cohorts reported elsewhere (Canfarotta et al., 2020).

**FIGURE 2:**
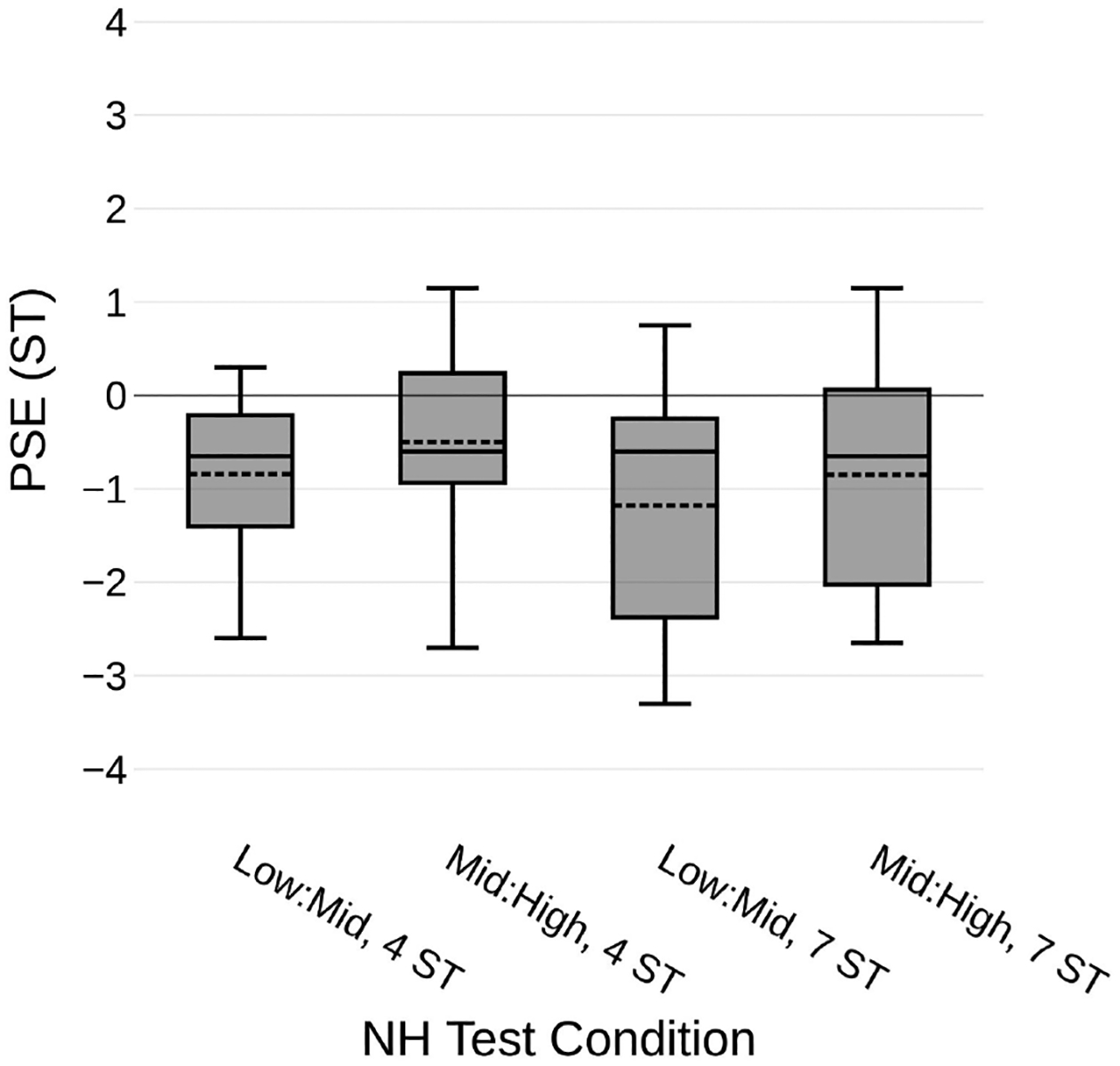
PSE for NH unprocessed condition. Pitch interval comparison results for NH listeners assessing intervals of 4 and 7 ST. The x-axis indicates the frequency ranges being compared (Low:Mid, Mid:High) and the size of the pitch interval (4 or 7 ST). The horizontal solid line represents the median, while the dashed line represents the mean. The y-axis shows the Point of Subjective Equality (PSE), indicating the actual semitone difference required for intervals in different frequency ranges to be perceived as equal. A PSE of 0 means intervals were perceived as equal when the actual semitone difference was identical. A negative PSE means the pitch interval in the higher frequency range (e.g. C7-G7) had to be smaller than the interval in the lower frequency range (e.g. E5-A5) to be perceived as equal. This indicates a relative perceptual overestimation of interval size at higher frequencies.

**FIGURE 3:**
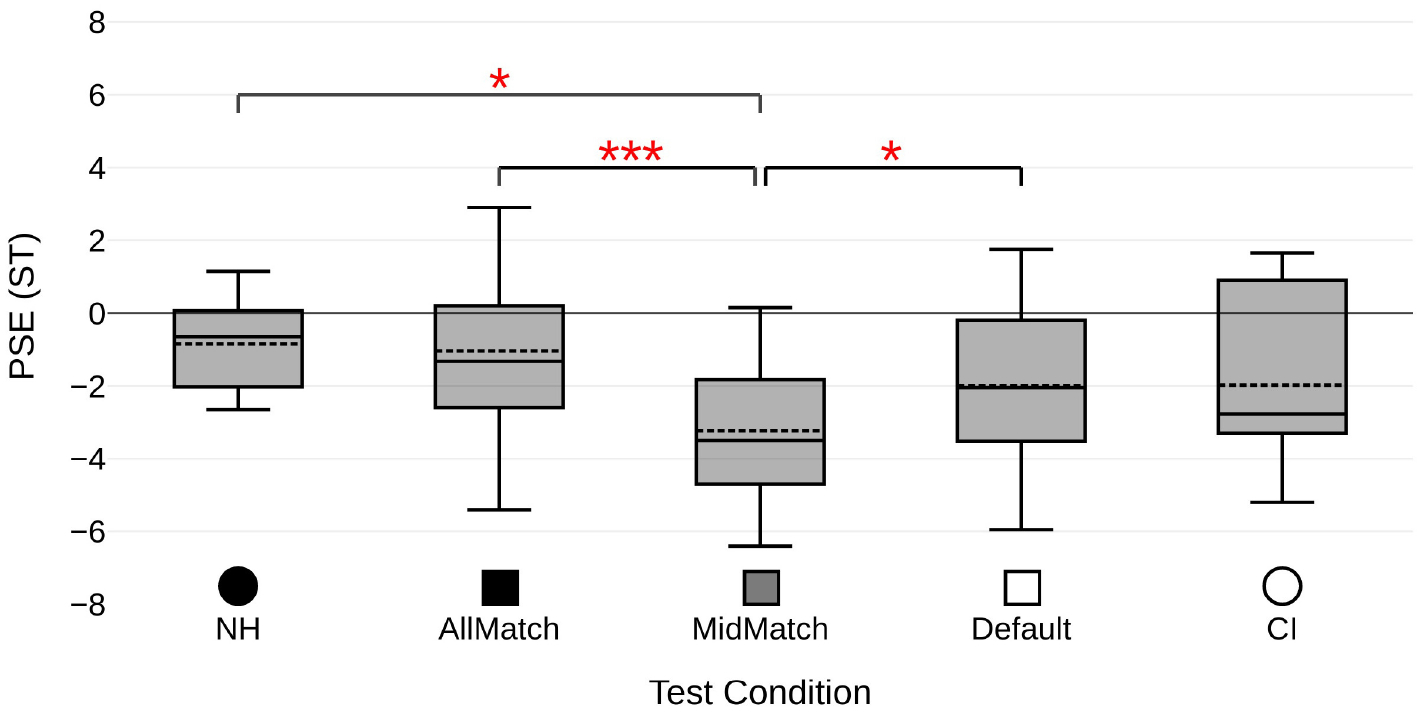
PSE in Mid:High range across five listening conditions. Pitch interval comparison results from five listening conditions, all using a 7 ST fixed pitch interval for reference and comparing the Mid:High frequency ranges. Box plots include both median in solid lines and mean in dashed lines. From left to right: NH listeners with unprocessed pure-tone stimuli, vocoded 24 mm array with all frequencies matched, vocoded 28 mm array with middle frequencies matched, vocoded 28 mm array with default frequencies, and CI recipients with default frequencies. *p < 0.05, ***p < 0.001.

The second step to create the vocoded stimuli was to apply a frequency allocation table to simulate either default or custom CI filterbank settings, which yielded the following three conditions: (1) Vocoded 28 mm Array with Default Frequencies (“Default”, Figure 1A), (2) Vocoded 28 mm Array with Middle Frequencies Matched (“MidMatch”, Figure 1B), and (3) Vocoded 24 mm Array with All Frequencies Matched (“AllMatch”, Figure 1C).

The Default setting (Figure 1A) used a frequency range of 70–8,500 Hz, logarithmically divided into 12 channels, and was modeled after the manufacturer’s default frequency allocation table (LogFS).

The MidMatch (Figure 1B) applied a strict match of center frequencies to the mid-frequency range (950–4,000 Hz) and the remaining frequency ranges were then redistributed across the most apical and basal electrodes (70–950 Hz, 4,000–8,500 Hz). This approach attempted to maintain audibility across the entire frequency range utilized by the Default setting, while also maintaining absolute pitch interval integrity where feasible.

The All Match utilized a strictly CT-based approach (Figure 1C), which matched channel center frequencies to all of the apical and mid-array electrode contact locations (<4,000 Hz). To avoid deactivating electrodes that were located above the bandwidth limit of the software (8,500 Hz), a logarithmic redistribution was applied to the highest frequencies (>4,000 Hz) to make best use of available remaining electrodes.

CI frequency allocation tables (horizontal bars) with electrode locations (circles). A: The 28 mm electrode array with the CI manufacturer’s default logarithmic filterbank (LogFS, “Default”). B: The 28 mm electrode array with middle frequencies (950–4,000 Hz) matched (“MidMatch”). C: The 24 mm electrode array with all frequencies (<4,000 Hz) matched (“AllMatch”).

#### Standardization of sound levels for participant assessment

Given the diversity in computer audio configurations used by participants to complete the study, variability in presentation levels and frequency response was a significant challenge. To ensure audibility of task stimuli for all listeners, we implemented a personalized level standardization process. Participants adjusted five sliders (±30 dB range) to equalize the loudness of pure tones at 125, 250, 500, 2,000, and 7,352 Hz, creating a “most comfortable” listening level profile across the frequency spectrum.

After adjusting each slider independently, participants could replay the five tones as a sequence to check they were equivalent in loudness before saving. The adjusted levels were used to construct a participant-specific frequency response profile via linear interpolation in MATLAB, applied to all stimuli, including vocoded ones, to ensure consistent and optimal listening levels across different computer audio hardware setups.

### Statistical analysis

All statistical analyses were completed using JASP software (JASP, Version 0.18.3; Amsterdam, Netherlands).

#### Demographics: age and training

Analysis of the demographic variables involved descriptive statistics to outline the sample distributions, Shapiro-Wilk test to detect non-normal distribution, Kruskal-Wallis test for differences between groups, Dunn *post-hoc* comparisons, and Spearman’s rho correlation coefficients for comparison with the dependent variable. One participant in the study, who used bilateral CIs, completed the activities twice, once with each device; for this individual, the data from each ear were analyzed separately.

#### Normal hearing with unprocessed stimuli

The primary outcome measure was the point of subjective equality (PSE) for pitch intervals. The normal hearing (NH) cohort was analyzed through use of two-way repeated-measures ANOVA to compare means across test conditions; we then performed a Wilcoxon’s matched pairs signed-rank tests to identify significant pairs as described by Nayak and Hazra (2011). To assess differences from a zero PSE, which indicates veridical or accurate perception, data were pooled across factors that did not show significant effects and one-sample *t*-tests were completed.

#### Normal hearing with vocoded stimuli

The parametric data from the NH cohort listening to vocoded stimuli were analyzed with a one-way repeated-measures ANOVA. As above, to further investigate the significant difference identified by the ANOVA, we then performed a Wilcoxon’s matched pairs signed-rank test to identify significant pairs (Nayak and Hazra, 2011). For the specific comparison between NH (unprocessed) and vocoded AllMatch, where the variances were not equivalent as determined by the Brown-Forsythe test, the Welch test was used. A Greenhouse-Geisser correction was applied to the ANOVA since the assumption of sphericity within the data was violated.

#### CI-mediated listening

An independent samples *t*-test was used to compare the CI group and the vocoded Default condition. Additionally, we conducted a one-sample *t*-test to compare the results to a zero PSE, indicating accurate pitch perception, for the CI group as was also completed for the NH group.

## Results

### Demographics

As expected, there were significant differences in age between the NH listeners and the CI recipients, evidenced by the significant main effect of group on age (H(2) = 21.0, *p* < 0.001) and the significant contrast between the CI group and the NH groups (CI and NH: *p* < 0.001, CI and Voc: *p* = 0.002) but not between the two NH groups (NH and Voc: *p* = 0.084). There were no significant differences between the groups in terms of duration of musical training (H(2) = 0.138, *p* = 0.933).

### Normal hearing with unprocessed stimuli

For NH participants listening to unprocessed stimuli, fixed pitch intervals included both 4 ST and 7 ST across the Low:Mid and Mid:High frequency regions (Figure 2). A two-way repeated-measures ANOVA showed a significant effect of interval size [*F*_(1, 14)_ = 8.42, *p* = 0.012], but no effect of frequency range [*F*_(1, 14)_ = 2.49, *p* = 0.137] and no significant interaction [*F*_(1, 14)_ = 0.001, *p* = 0.973]. NH listeners judged lower frequency intervals as slightly larger than higher ones. When pooled across (non-significant) frequency range, the mean PSE with the 4 ST fixed interval was−0.7 ST, which was significantly different from zero (*t*_(14)_ = −2.84, p = 0.013) and −1.0 ST with the 7 ST fixed interval, which was also significantly different from zero (*t*_(14)_ = −3.57, *p* = 0.003). Despite this small bias toward perceiving higher-frequency intervals as smaller than lower-frequency intervals, the results show that NH listeners are able to perform the task and demonstrate near-veridical perception of intervals across different frequency regions.

### Normal hearing with vocoded stimuli

For the vocoded conditions, the AllMatch condition, which is most similar to the NH unprocessed condition, yielded the most accurate pitch interval perception, with a mean PSE of −1.04 ST (SD: ±2.29). A between-subjects comparison of these data with the corresponding condition from the NH group with the unprocessed stimuli (Mid: High frequency range, 7 ST) showed no significant difference (*t*_22.8_ = −0.298, *p* = 0.768), as illustrated by the two leftmost boxplots in Figure 3.

The vocoded MidMatch condition resulted in the least accurate performance, as evidenced by the largest average (absolute) PSE value of −3.23 ST (SD: ±1.94). Lastly, the vocoded Default map demonstrated marginally more accurate perception, with a mean PSE value of −1.99 ST (SD: ±2.28). A within-subjects comparison of the means from the three vocoded conditions confirmed a significant effect of condition on the PSE (*F*_1.3,19.9_ = 12.0, *p* = 0.001). Paired comparisons between the three conditions found no significant difference between the Default and AllMatch conditions (*z* = 1.40, *p* = 0.175), but did find a significant difference between the Default and MidMatch conditions (*z* = −2.10, *p* = 0.038) and between the AllMatch and MidMatch conditions (*z* = 3.52, *p* < 0.001).

### CI-mediated listening

Participants with CIs were tested using pure-tone stimuli and with the same test parameters as the vocoded cohort (Mid:High frequency range, 7 ST reference interval). All CI recipients used the manufacturer’s default frequency allocation table (LogFS, 70–8,500 Hz), as shown in Figure 1A, although the physical electrode locations varied by patient. Mean results from this group, shown Figure 3 on the far right (CI), were very similar to the vocoded condition that also utilized the Default frequency allocation table (*t*_24_ = 0.015, *p* = 0.988). The mean PSE was −1.98 ST (SD: ±2.37), which was significantly different from zero (*t*_9_ = −2.64, *p* = 0.027). These data demonstrate that CI recipients could successfully complete the task. It is also encouraging that their results were very similar to those from the vocoded condition designed to approximate the default CI map.

### Summary of key findings

**FIGURE 4:**
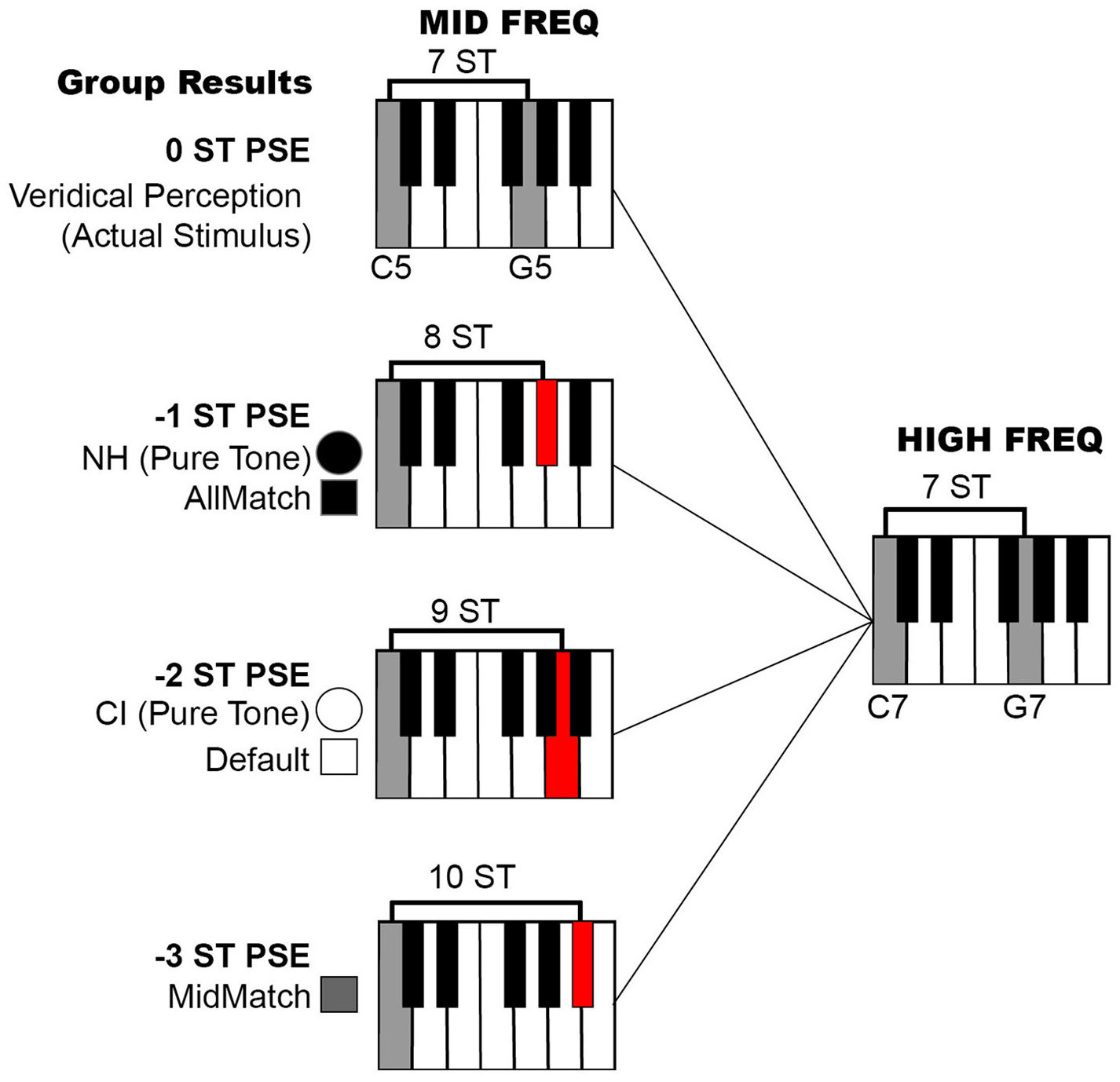
Piano illustration of PSE results. The test results from the five listening conditions noted in Figure 3, depicted on a piano scale to assist with interpretation. The top row represents a 7 ST interval in the mid-frequency range (C5-G5) matching a 7 ST interval in the high-frequency range (C7-G7), representing an ideal PSE of 0 ST (veridical pitch interval perception). The second row displays the two conditions yielding the most accurate results, with a PSE around −1 ST: the NH and vocoded AllMatch groups. The third row presents results from the CI and vocoded Default groups, with a PSE around −2 ST, and the bottom row shows data from the vocoded MidMatch group, which had a PSE around −3 ST. PSE, point of subjective equality; ST, semitone.

Taken together, the PSE results reveal distinct trends in pitch interval perception across the listening conditions, with the NH and AllMatch groups showing the most accurate performance, both approaching a PSE of −1 ST. CI recipients, as well as the Default group, exhibited a slightly larger but still comparable shift at around−2 ST, while the MidMatch group showed the greatest deviation at approximately −3 ST. These differences are illustrated on a piano scale in Figure 4, to aid the reader’s understanding of the relative accuracy in pitch perception across conditions. These findings suggest that the alignment of frequency maps, particularly in CI programming, plays a key role in maintaining more accurate pitch perception.

### Impact of age and music training

To examine the impact of age and musical training on performance, a grand average PSE value was generated for each participant by averaging their test results. No significant linear relationship was found between pitch interval task performance and either years of musical training (Spearman rho = −0.153, *p* = 0.346) or age (Spearman rho = −0.254; *p* = 0.114). Each cohort (NH, Voc, and CI) was also tested individually; the correlation coefficients found for age and musical training were again not significant (*p* > 0.05 in all cases), ranging from rho = −0.399 to rho = 0.360.

## Discussion

This study examined pitch interval perception across different listener groups, focusing on the comparison between normal-hearing (NH) individuals under various auditory conditions and CI recipients. Specifically, it assessed how different frequency allocation strategies, simulated through vocoded stimuli, impact pitch interval perception in NH listeners and compared these findings to the perceptual experiences of actual CI users. Additionally, the study investigated the role of age and musical training in shaping performance across both NH and CI groups, offering insights into the factors that may influence auditory processing in these populations.

Significant age differences were found between NH listeners and CI recipients, but no significant differences were observed in the duration of musical training among groups. While we cannot rule out age as a contributing factor to differences between groups, the influence of musical training on the tested perceptual tasks is consistent across groups. This points to the robustness of the perceptual task across different listener backgrounds, which fulfills an important foundational need for future clinical implementation of this task.

NH listeners demonstrated near-veridical perception of pitch interval estimation when evaluating unprocessed stimuli, which demonstrates that NH listeners can generally perform this task that aims to assess pitch perception across different frequency regions. There was a slight, yet significant, bias for NH listeners to perceive lower frequency intervals as larger than high-frequency intervals. In previous studies of pitch intervals, this issue was not examined, and the topic deserves further study.

Among different vocoded conditions, the condition with all frequencies matched (AllMatch) showed the most accurate pitch interval perception, closely aligning with the NH listener results when listening to unprocessed stimuli. This finding underscores the potential improvements that could be achieved for patients with CI(s) when using a frequency allocation table that is more closely aligned with the electrode position across all available frequencies. This supports continued investigations into anatomy-based fitting approaches that utilize post-operative imaging for individual patients (e.g., Jiam et al., 2019b; Alahmadi et al., 2024; Kurz et al., 2023) for personalized guidance with CI programming, with the goal of improving the pitch-place mismatch.

Significant differences were observed within the vocoded conditions; notably, the middle frequencies matched condition (MidMatch) yielded the least accurate perception, suggesting that frequency allocation tables that use this type of approach may significantly reduce pitch perception accuracy when comparing Mid:High frequency ranges. However, this result may not directly translate to clinical outcomes, as real-world performance can be influenced by factors beyond those tested in this study. Due to the lack of audibility of the low frequency range in the AllMatch condition, this study did not include a Low:High or Low:Mid option for vocoded conditions.

Finally, when evaluating CI recipients’ actual performance, who were all using default frequency mapping, their pitch interval perception was very similar to that of NH listeners using a vocoded condition that approximated the default CI map. This suggests that a revised approach to CI programming (i.e., anatomy-based fitting) could allow clinicians to achieve close-to-normal pitch, as others have previously suggested using other methods (Heitkötter et al., 2024).

Taken together, these results demonstrate proof of concept that this task allows for an assessment of relative pitch interval perception in people with normal hearing and those with CI at a group level. However, additional larger-scale studies will be needed to determine whether this could be used at an individual level in a clinical setting for CI recipients.

### Implications for CI programming

The findings from this study may offer potential implications for CI programming in clinical environments. These insights pave the way for enhancing auditory outcomes for CI recipients, particularly in the realms of music perception and complex auditory scene analysis (Drennan and Rubinstein, 2008).

Improved pitch perception accuracy observed in NH listeners with the AllMatch vocoded conditions underscores the potential in improving pitch-place matching across frequencies in CI programming approaches (Jiam et al., 2019b; Noble et al., 2014; Svirsky et al., 2004). This suggests that CI programming could be significantly improved by ensuring that frequency allocation closely aligns with the individual’s cochlear anatomy. Clinicians might consider adopting more refined and personalized frequency maps to better simulate natural hearing; for example, studies in recent years have begun to investigate anatomy-based fitting as an approach that could help CI users achieve more natural hearing experiences (Dillon et al., 2023; Jiam et al., 2021; Kurz et al., 2023; Noble et al., 2014).

It is also important to consider the variation in performance across different vocoded conditions; similar variation is observed in CI recipients due to factors such as variability in cochlear size and surgical electrode array placement (Alahmadi et al., 2024; Spiegel et al., 2022), variability in neural survival, and associated variability in the electrode-neuron interface (Jahn and Arenberg, 2020), among other factors. Results from the default vocoded condition, which mirrors standard CI programming parameters, when compared to other vocoded conditions completed by NH listeners—representing personalized approaches to CI programming—indicate that a one-size-fits-all approach may contribute to poorer individual outcomes. This is because utilizing the default frequency allocation tables may not always lead to optimal outcomes (Holden et al., 2013; Jiam et al., 2019b; Noble et al., 2014). These findings underscore the importance of considering individual factors such as electrode placement, cochlear duct length, and neural interface in CI programming to enhance performance. Tailoring the CI frequency mapping to fit the unique auditory profile of each user could potentially improve speech understanding and environmental sound perception.

Finally, the ability of NH listeners to accurately perceive pitch intervals under simulated CI conditions indicates that, with personalized programming that take into account individual needs, CI recipients could potentially improve their music appreciation (Drennan and Rubinstein, 2008; Drennan et al., 2015; Gfeller et al., 2008). An improvement in music perception could enrich recreational activities and social interactions, contributing to greater overall life satisfaction (Gfeller et al., 2002).

### Limitations and future directions

The study utilized acute exposure to different vocoded conditions, which primarily reflects the immediate effects of auditory stimuli under controlled conditions. While this approach provides valuable insights, it may not fully capture the long-term adaptation and learning effects that chronic exposure would produce, thereby limiting the generalizability of our findings to real-world settings (Gfeller et al., 2008; Svirsky et al., 2004). Cochlear implant recipients often experience significant changes in their perceptual abilities as they adapt to their devices over time, suggesting that our results may not entirely represent the potential improvements or challenges CI recipients might encounter during everyday, long-term use (Fu and Galvin, 2007; Looi et al., 2012).

Adaptation to different methods of listening (e.g., CIs or vocoded stimuli) highlights an opportunity for implementing auditory training programs (Henderson Sabes and Sweetow, 2007; Saunders et al., 2016; Sweetow and Sabes, 2006). Such programs, particularly those focusing on pitch discrimination and music appreciation, could substantially enhance not only the enjoyment of music but also overall communication effectiveness in complex listening environments (Drennan and Rubinstein, 2008; Gfeller et al., 2015; Jiam et al., 2019a; Looi et al., 2012). Future studies should explore the long-term benefits of these training programs, assessing their impact on both speech and music perception after extended use.

Another limitation is that only one CI manufacturer was represented in the study. This decision, made to control for device-specific variables, nonetheless restricts the broader applicability of our findings. Different CI manufacturers offer varying technologies and device settings, which can significantly influence the number and quality of distinct pitch percepts provided across the electrode array (Holden et al., 2016; Noble et al., 2014). Further, current CI systems limit the upper frequency range to approximately 8,500 Hz. Expanding this range—potentially to 20,000 Hz, as clinicians have recommended—could introduce tools for clinicians to reduce the spectral mismatch that occurs above 4,000 Hz, making frequency allocation more accurate across all electrodes. Consequently, these findings may not be generalizable to all CI technologies, particularly those with differing approaches to frequency allocation and spectral resolution (Jiam et al., 2019b). Future research should include a range of CI devices from multiple manufacturers, especially if those devices could include broader frequency ranges, to determine whether the observed effects are consistent across different technologies.

Additionally, the study only tested vocoded conditions in the mid-frequency range, as the AllMatch condition did not extend below approximately 500 Hz. This presents a limitation, as a substantial portion of music (Gilbert et al., 2019), as well as critical aspects of speech perception (Byrne et al., 1994), predominantly occurs in the low frequencies. Without testing the low-frequency range, our understanding of pitch perception in these conditions remains somewhat limited. Moreover, comparing participants with and without access to fine structure cues during the task could provide deeper insights into the mechanisms underlying pitch perception among CI recipients. Future studies should aim to evaluate a broader — and lower — frequency range to capture a more comprehensive picture of auditory perception in CI recipients.

Finally, longitudinal studies that monitor changes in pitch perception after individuals adapt to new programming conditions would be invaluable. Such studies would reflect auditory plasticity and help address the limitations of our current approach by providing insights into how CI recipients adapt to changes in frequency mapping over time. Additionally, future research should evaluate a wider array of electrode configurations to better understand how variations in spectral resolution impact pitch perception across different CI manufacturers’ devices.

## Figures and Tables

**TABLE 1:** Demographic information of the CI recipients, displayed on a per-ear basis.

ID #	Ear	Age (yrs)	Gender	Duration CI use (yrs)	Duration musical training (yrs)	Etiology	Implant model	Electrode array	Processing strategy	CNC (%)
1	R	71	F	6.3	0	Idiopathic	Concert	FLEX28	FS4-p	66
2	R	58.1	F	8.2	15	Idiopathic	Concert	FLEX28	FS4-p	66
2	L	58.1	F	10.5	15	Idiopathic	Concert	FLEX28	FS4-p	60
3	R	44.2	M	9.7	4	Genetic	Concert	Medium	FS4	72
4	R	60.2	F	6.9	1	Genetic	Concert	FLEX28	HDCIS	66
5	R	76.2	M	4	5	Idiopathic	Synchrony	FLEX28	FS4-p	34
6	L	64.1	M	2.4	35	Noise Exposure	Synchrony	FLEX28	FS4-p	74
7	R	40	M	4.9	22	Genetic	Synchrony	FLEX28	FS4	Unk.
8	R	62.6	M	1.9	20	Idiopathic	Synchrony	FLEX24	FS4-p	42
9	R	40.1	M	4	0	Genetic	Synchrony	FLEX28	FS4	Unk.

ID, participant identification number; CNC, consonant-nucleus-consonant word recognition test scored by percentage of words correct on a 50-word list; F, female; FLEX24 and medium, 24 mm electrode array; FLEX28: 28 mm electrode array; FS4 and FS4-p, fine structure processing (-p indicates parallel signal processing); HDCIS, high-definition continuous interleaved sampling; L, left; M, male; Unk., unknown; R, right, yrs, years.

## Data Availability

The datasets presented in this study can be found in online repositories. The names of the repository/repositories and accession number(s) can be found at: https://doi.org/10.5061/dryad.dfn2z359d.
